# Considerations for Subgroups and Phenocopies in Complex Disease Genetics

**DOI:** 10.1371/journal.pone.0071614

**Published:** 2013-08-20

**Authors:** Ryan Ramanujam, S. Ramanujam, Jan Hillert

**Affiliations:** 1 Department of Clinical Neuroscience, Karolinska Institutet at Karolinska University Hospital Solna, Stockholm, Sweden; 2 The Peter J. Tobin College of Business, St. John’s University, New York, New York, United States of America; Tor Vergata University of Rome, Italy

## Abstract

The number of identified genetic variants associated to complex disease cannot fully explain heritability. This may be partially due to more complicated patterns of predisposition than previously suspected. Diseases such as multiple sclerosis (MS) may consist of multiple disease causing mechanisms, each comprised of several elements. We describe how the effect of subgroups can be calculated using the standard association measurement odds ratio, which is then manipulated to provide a formula for the true underlying association present within the subgroup. This is sensitive to the initial minor allele frequencies present in both cases and the subgroup of patients. The methodology is then extended to the χ^2^ statistic, for two related scenarios. First, to determine the true χ^2^ when phenocopies or disease subtypes reduce association and are reclassified as controls when calculating statistics. Here, the χ^2^ is given by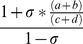, or 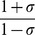 for equal numbers of cases and controls. Second, when subgroups corresponding to heterogeneity mask the true effect size, but no reclassification is made. Here, the proportion increase in total sample size required to attain the same χ^2^ statistic as the subgroup is given as 
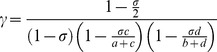
, and a python script to calculate and plot this value is provided at kirc.se. Practical examples show how in a study of modest size (1000 cases and 1000 controls), a non-significant SNP may exceed genome-wide significance when corresponding to a subgroup of 20% of cases, and may occur in heterozygous form in all cases. This methodology may explain the modest association found in diseases such as MS wherein heterogeneity confounds straightforward measurement of association.

## Introduction

Advances in genotyping technology have allowed for large scale genome wide association studies using up to millions of SNPs in cohorts of several thousand cases and controls. The data produced contains a wealth of information, which often results in the discovery of new gene associations with a given disease. However, despite the tremendous advances in technology, meta-analyses of large cohorts are required to identify new disease associated genes, which often have small effect sizes.

Complex diseases are defined as those which have multiple genetic components as well as environmental interaction [1]. Often this underlying genetic predisposition causes no disease manifestation for many years, until either a threshold of environmental exposure or a triggering event occurs, after which the disease begins. Frequently, complex diseases such as rheumatoid arthritis (RA) are referred to as “syndromes of diseases” which have similar phenotypic manifestations with at least partly unrelated disease pathogenesis, evidenced by cases both with and without autoantibodies present [2].

**Figure 1:**
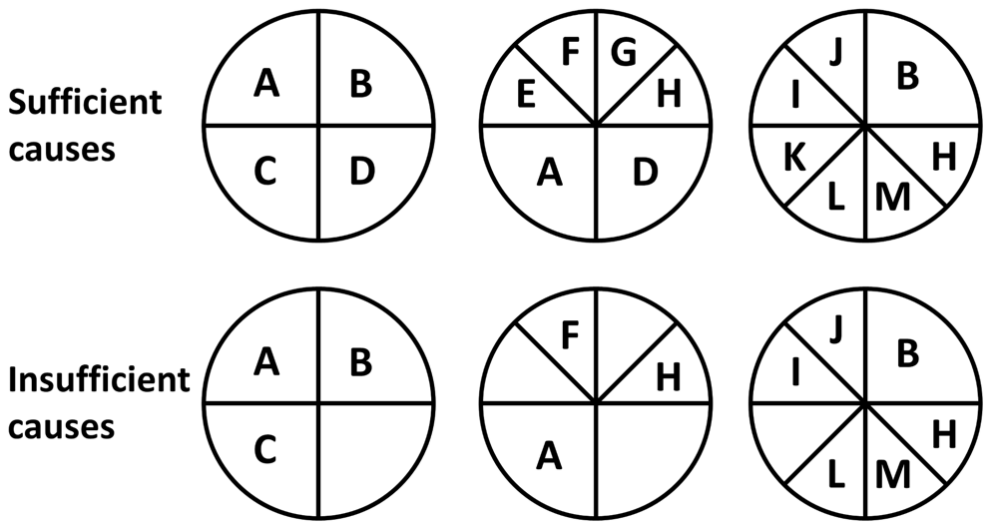
Pie model of sufficient causes for complex disease. Complex diseases may be multiple disorders with similar phenotypic manifestations, or a disorder with multiple genetic causes (subclasses). Each of the subclasses may be a result of combinations of similar, or unique, predisposing genes.

Multiple sclerosis (MS) is a complex autoimmune disorder which may have either different disease mechanisms and/or genetic background; that is, the genetic factors influencing an individual’s predisposition may vary. The Rothman pie model of sufficient causes postulates that subgroups of disease may exist within “pies” of a predetermined number of genetic and environmental factors [3]. The presence of all such factors represents a sufficient cause, and in individuals with all pieces of a single pie, disease develops (Figure 1). The low effect sizes of many genes outside the major histocompatibility complex (MHC) in these diseases, despite estimates of only a handful of genes required to confer disease predisposition in twins studies [4], may indicate that the pie model is correct.

The existence of genetic subgroups of disease likely confounds identification of genes contributing to the predisposition of complex disorders. A simulation study using reasonable values for samples size, effect size and allele frequencies estimated the effect of subgroups and modifier gene on detection thresholds [5]. It determined that studies 1000 cases and 1000 controls typically have 80% power to detect allele odds ratios (OR) of 1.7 in the presence of such confounding effects, far beyond the effect size observed for all genes outside the MHC in most complex disorders.

A common method for improving detection of genetic association is to stratify disease samples based on clinical characteristics. For example, in RA patients with the presence of antibodies to citrullinated peptide antigens (ACPA+) display clear differences in association from ACPA- [6]. In MS, disease subtype may include disease course. However, for measures without concrete and unchanging characteristics, such as severity, these may be inexact and alter over time. A further complication is that genetic subgroups may present a wide range of clinical characteristics, particularly in disease with changing course over time.

The failure of genome-wide association studies (GWAS) to identify new variants with strong effects on disease predisposition has led to the search for “missing heritability”. It has been proposed that detection of more variants and/or rare variants may be useful, particularly by conducting large scale sequencing of patient samples [7]. Here, we examine further issues pertaining to the presence of genetic subgroups on the OR for association studies in complex disorders, with application to autoimmune genetics. In particular, we derive the true OR for a subgroup of disease based on the proportions of the subgroup within all disease samples. We extend this methodology to the related issue of the presence of phenocopies or distinct disease subtypes, which complicate the calculation of χ^2^ statistics, and derive a function to explain the true association present. Finally, we present a function to determine sample sizes required to attain the association contained within the subgroup only.

## Methods and Results

Within a syndrome of diseases or one with several genetically distinct predispositions, an effect strong enough to alter the OR of the total sample may have a much higher effect in the genetic subgroup in which it is a predisposing element. A basic assumption of this relationship is that allele frequencies are altered within one or more subgroups, and remain similar to controls in “non-affected” subgroups.

### Subgroup Odds Ratios

**Figure 2:**
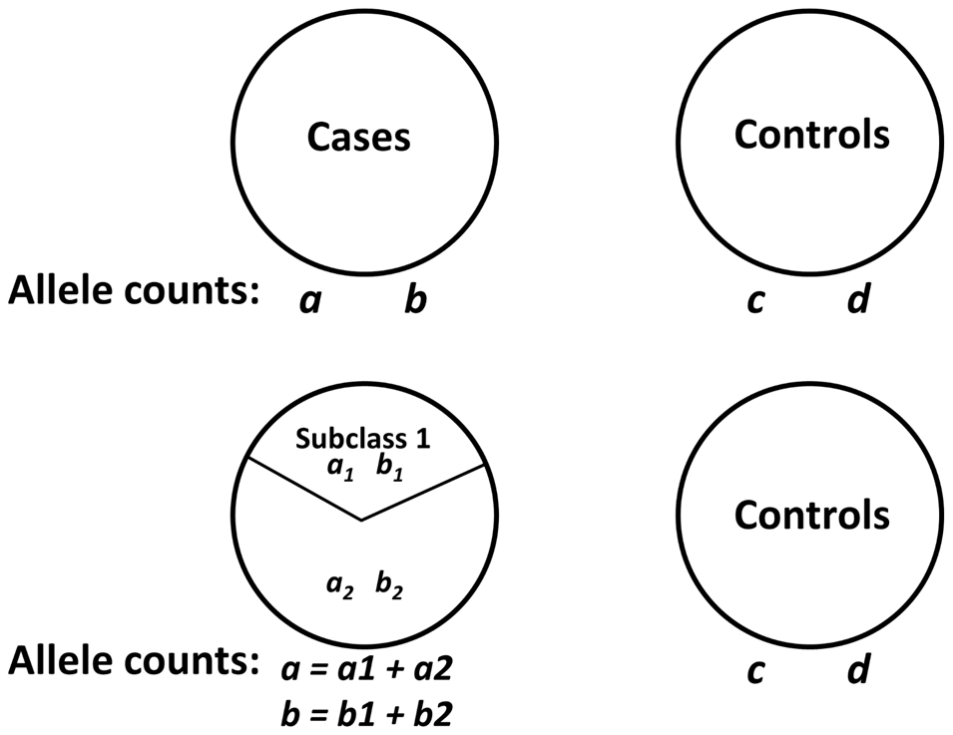
Allele frequency alterations due to the presence of a subgroup. The difference in allele frequencies for a SNP considered classically (top) and as a complex disorder with subgroups (below). In the example, a hypothetical subgroup denoted Subclass 1 contains allele counts *a_1_* and *b_1_*, while the rest of the cases contain allele counts *a_2_* and *b_2_*.

If a single nucleotide polymorphism (SNP) has a certain genotype (e.g. AA, Aa/aa, aa) in all cases of one of n genetically distinct subclasses of disease, the OR will reflect an overall regression to the population’s allele frequencies at that SNP. Assume that a SNP has allele counts in cases given by *a* and *b*, allele counts in controls given by *c* and *d*, and that a subclass exists within the cases with allele counts *a_1_* and *b_1_* such that 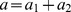 and 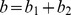 (Figure 2).

The underestimation in OR can be measured as a ratio of the “true” OR of the subclass to the OR of the entire group
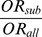
where the standard 2x2 contingency table given in Table 1 becomes that in Table 2. and 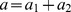 and 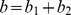. The OR_sub_ is defined by 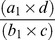 and OR_all_ by 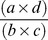 this becomes 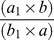 and, which is equivalent to

**Table 1:** 2×2 contingency table of allele counts.

	Allele 1	Allele 2
**Cases**	*a*	*b*
**Controls**	*c*	*d*

**Table 2:** 2×2 contingency table of allele counts given the presence of a subclass.

	Allele 1	Allele 2
**Cases**	*a_1_*	*b_1_*
**Controls**	*c*	*d*



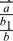



Since 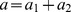 and 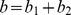, this can also be represented as



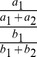
(1)


This illustrates that the OR has been underestimated by a factor relying solely on the proportion of one allele present in the subclass to the proportion of the other allele present in the subclass. This can be termed the error factor.

**Figure 3:**
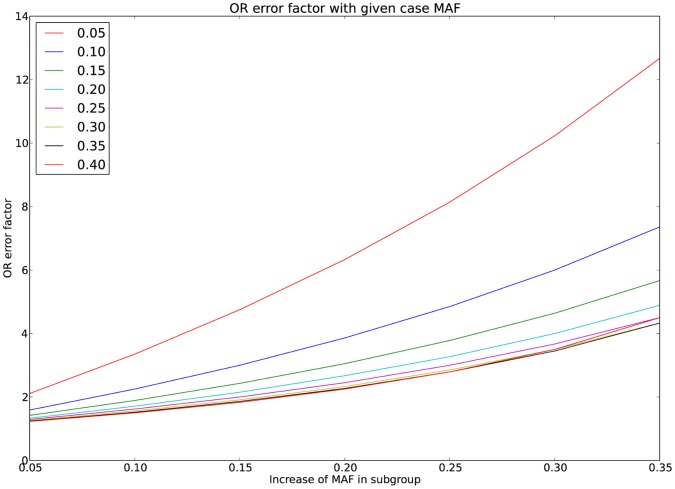
Error factor in ORs. OR error factor present with various minor allele frequencies. Each curve represents the range of OR error factors due to subgroups for a given MAF (minor allele frequency) observed in all cases. The Y-axis indicates the OR error factor corresponding to an increase in the subgroup MAF (as compared with all cases) given by the X-axis. For example, in the second curve corresponding to a MAF of 0.10 in cases, a subgroup with an increase of MAF of 0.20 (or 0.30 MAF) would have an OR in that subgroup approximately four times that reported for the overall group.

This effect of error factor on OR is plotted for various minor allele frequencies separately in Figure 3. Each curve corresponds to a SNP with a MAF of a given value in cases. If there is an increase in the MAF in a subgroup (x-axis), the OR error factor increases correspondingly along the y-axis.

### Phenocopies

A second and related application of this rationale is to determine the error in association measured due to the presence of disease subtypes or non-genetic causes of disease, usually denominated phenocopies. These subsets of disease may be distinguishable from other clinical groups and contain a distinct etiology or are a different disorder.

Phenocopies have a measureable effect on the χ^2^ statistic calculated. To investigate the potential for omitting relevant SNPs due to this (Type II error), we assume that some proportion of cases are separate disease subtypes or not genetic in nature, and call this term σ. In order to estimate the true allele frequencies of a given SNP in the relevant cases, we remove the phenocopies, and add them to controls with the previously determined control frequency. We recalculate the allele frequency that was present in the remaining cases, and can determine the χ^2^ value which corresponds to the true frequency of the SNP in these cases. We have original observed and expected allele counts as follows: observed as given previously in Table 1 and expected as given in Table 3.

**Table 3:** Expected allele counts in χ^2^ statistic calculation.

	Allele 1	Allele 2
**Cases**	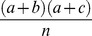	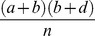
**Controls**	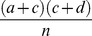	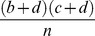
	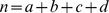	

To find the ratio of error in χ^2^ values, we state that the χ^2^ value of the new allele distribution is χ^2^_n_. The ratio 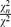 provides a measure of the relative error in strength of association as well as a means to calculate the true association based on the allele counts and the proportion of included cases. Since σ is a proportion, the number of *a* and *b* removed and added to *c* and *d* is given by the number to remove, 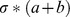, multiplied by the frequency of the allele in controls: 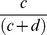 or 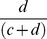
which becomes
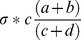
and



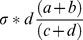



The new observed values to calculate χ^2^_n_ can be denoted *a_n_*, *b_n_*, *c_n_* and *d_n_* and are given in Table 4.

**Table 4:** Adjusted observed values when phenocopies are reclassified as controls.

a_n_	b_n_
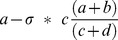	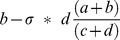
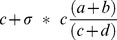	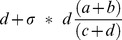
c_n_	d_n_

The function 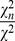 can be calculated using the standard formula 
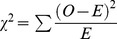
 for each term of the original data.



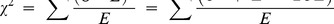
which is 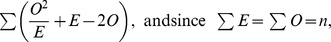




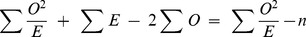



Entering each term for observed and expected:



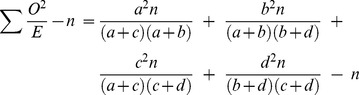
which simplifies to:



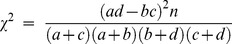



For the new data, the value of the formula is modified with the new observed values *a_n_*, *b_n_*, *c_n_* and *d_n_*:



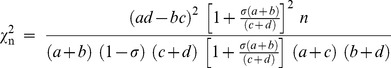



The ratio 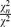 therefore simplifies to the function
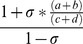
(2)which shows that the proportion increase in χ^2^ value is determined by the value of 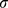 and the original allele frequencies. This formula can be altered as follows:



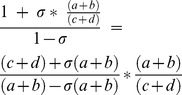



The first term is equal to 
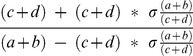
 which is 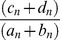.

This simplifies to
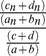
which yields the ratio of total controls to total cases in the new data, normalized by the same quantity in the original data.

**Figure 4:**
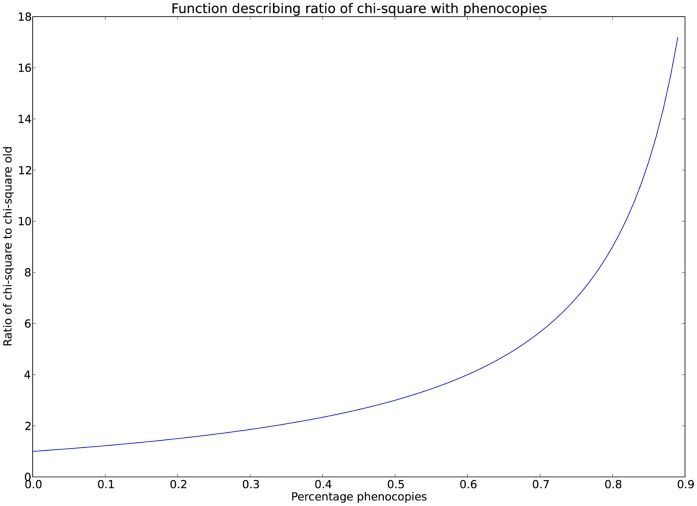
χ^2^ ratio function. The function 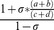 describes the ratio of the χ^2^ value with only genetic cases to that of the original data. The plot above is for equal numbers of cases and controls, so that 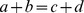, making the function 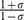. For example, with equal cases and controls, if 20% of cases have non-genetic causes, the new χ^2^ statistic when removing these will be 1.5 times that reported.

The function 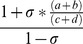 can be simplified for equal numbers of cases and controls, such that 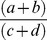 equals 1. The function then becomes 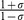 and is plotted in Figure 4.

The presence of phenocopies overstates the impact of the χ^2^ statistic for the core disease group, which exists with other subtypes as a proportion of overall cases. Thus, reclassification of cases not within a particular subtype to controls assumes a lack of disease predisposition, which is clearly not true for correctly diagnosed patients. Therefore, a more conservative approach for calculating required sample sizes will be employed.

### Sample Size in the Presence of Subgroups

Next we examine cohorts with only a particular subgroup, or sum of subgroups, associated to the disease at a particular locus. Consider a SNP which is weakly associated to the disease, but wherein only a minority of cases are contained within the subgroup exhibiting the association. In this situation, we preserve the coding of the proportion in the subgroup, σ, but do not add the samples removed from a′_n_ and b′_n_ to c′_n_ and d′_n_ as illustrated in Table 5.

**Table 5:** Adjusted observed values when cases not present in a subgroup are removed.

a′_n_	b′_n_
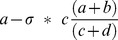	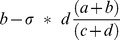
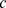	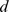
c′_n_	d′_n_

A new term, 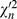, represents the χ^2^ statistic of the new table, namely the “true” χ^2^ statistic for only the subgroup in question, and is given by:



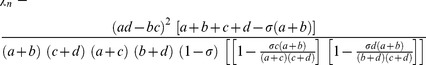
(3)


We now turn our attention to the relationship between the new statistic and the original one. In particular, how the original sample with given allele frequencies relates to the statistic of the subgroup. If allele frequencies remain fixed, how must the original sample size increase to report the same association?

In order to determine the increase in cases and controls required to replicate this statistic, without any reclassification of samples, a second 2x2 matrix is constructed to represent the new cohort size. A new variable, γ, is created which is a proportion by which the number of total samples must be increased in order to attain the χ^2^ statistic of the associated subgroup. Therefore, each term in the new matrix will have allele counts multiplied by γ (Table 6).

**Table 6:** 2×2 contingency table for samples increased by a factor of γ.

a_r_	b_r_
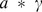	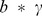
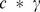	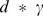
c_r_	d_r_


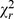
 is the term for this χ^2^ statistic, and is given as follows:



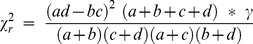
(4)


In order to determine sample sizes required, this must be equal to 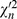, and thereafter a function of γ and σ can be derived.



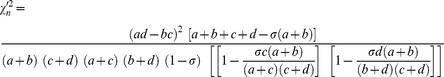





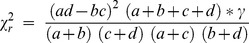



To decide for what increase in sample size 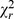 is equal to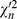, equations 1.3 and 1.4 are set to be equal and gamma is solved for.



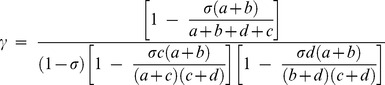
(5)


If a+b = c+d, i.e. for equal numbers of cases and controls, then the general equation for γ can be given as
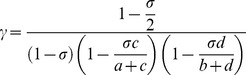
(6)


This represents the most generic case. It can be shown that γ increases as σ increases by taking the first derivative of equation 1.6 and showing it is positive. The factor 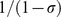 increases as σ increases and will be ignored in further discussions. The general case given in equation 1.5 involves taking the derivative with respect to σ and showing it is positive. In order to do this, let
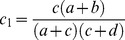


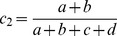


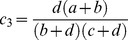



The derivative becomes 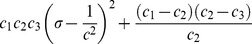. It turns out that 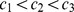 if ad-bc>0 or 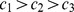 if ad-bc<0.

If ad-bc = 0 then 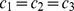. For this discussion it is necessary to assume the smallest of 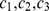 is <1. Otherwise, the denominator of γ becomes 0 in (0,1). Hence, the derivative is positive, showing γ is an increasing function of σ.

**Figure 5:**
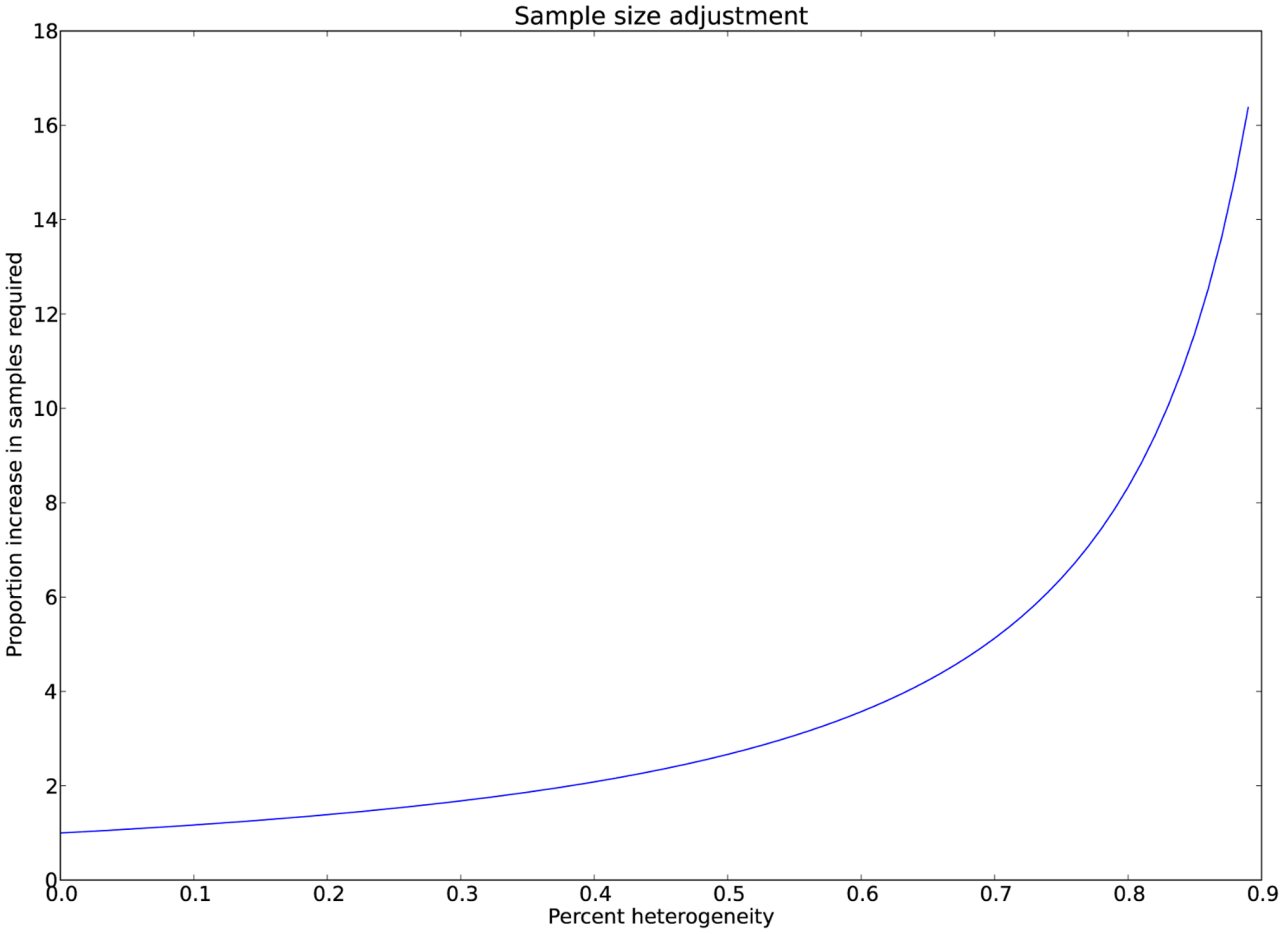
Sample size calculation due to heterogeneity. The increase in samples (γ) required as heterogeneity, defined by σ, increases for given values of a, b, c, d, when these values approach being equal. For example, if a = 1000, b = 2000, c = 1200 and d = 1800, and γ is estimated as 40% of cases not in the subgroup on which a given SNP acts, a relative increase in samples of 2.1 is needed to attain similar association statistics in the entire cohort as that of the underlying subgroup.

A python script to calculate γ for given values of a, b, c, d, and estimated σ via equation 1.5 is available at kirc.se/software/subgroups. The same script plots the function of γ for the range of σ as in Figure 5. As the proportion of heterogeneity for the subgroup increases (i.e. cases not contained within the subgroup) along with x-axis, a relative increase in samples with the original allele frequencies is required to achieve an identical χ^2^ statistic.

## Discussion

This paper explores the consequences to association studies of the possibility of SNPs to confer disease predisposition in a subset of patients only. Two scenarios have been explored, including subgroups in which cases not included are omitted, and an OR error is calculated based on the remaining cases and all controls. These calculations can be extended to determine sample sizes required to compensate for cases not in the subgroup. An additional examination of phenocopies, moved from cases to controls to determine allele frequencies, was conducted and a function relating the true χ^2^ statistic to the original calculation was derived.

The scenarios described, namely phenocopies and subgroups, are related and the determination of which to select for calculating the effect on OR, χ^2^ statistic or sample size is somewhat subjective. However, some examples utilizing overlapping clinical and genetic observations in both settings will be discussed, which may provide *a priori* expectations of how these scenarios might affect association studies. Practically, reclassification of phenocopies is less conservative than subgroup consideration, and is most suited with high certainty that a proportion of cases either have alternative causes which are non-genetic, or are disease subtypes displaying symptoms which may constitute a distinct disease or etiology. This scenario also provides a method to estimate the corresponding χ^2^ error factor provided only the proportion of heterogeneity, σ, for given allele frequencies, i.e. stratified sample sets.

In myasthenia gravis (MG), approximately 10–15% of patients display thymomas, which typically predates the disease and is considered to cause the symptoms [8]. Strictly speaking, thymoma in MG is not non-genetic as thymomas display many genetic associations [9] which are likely to predispose individuals to the cause of thymoma, possibly due to virus [10]. However, due to the distinct alternative cause of antigen immunization causing symptoms, these patients could be reclassified as controls to determine the true χ^2^ statistic for core, non-thymomatous MG. Assuming 15% thymoma, Equation 1.2 with equal numbers of samples would yield a χ^2^ error factor of 1.35.

In RA, ACPA+ disease appears to differ from ACPA-, with independent analysis of each group yielding different ORs across three independent cohorts [6]. Although not consistently higher in ACPA+ RA, notable associations such as SNPs in *PTPN22* (OR = 1.74 ACPA+, 1.23 ACPA-) and *TRAF1-C5* (OR = 1.32 ACPA+, 1.08 ACPA-) have increased effects in ACPA+ RA, and the authors consider this heterogeneity to denote a separate disease subtype. Given this, it may be reasonable to reclassify ACPA- cases with controls to enhance detection of variants that act only within the ACPA+ group. It is not possible to confirm if the ratio of χ^2^ error conforms to Equation 1.2 in the published report, since aggregation of ACPA- and healthy controls was not conducted. However, doing so given reported rates of 60% ACPA+ patients [11] would result in a χ^2^ error factor of 2.33. Simply put, failing to stratify on ACPA status could reduce the χ^2^ statistic in the ACPA+ group to less than half of that which might be obtained otherwise.

When a disease subtype is not suspected, or a common disease etiology cannot be ruled out, the subgrouping scenario without reclassification may be more appropriate. The effects of subgroups are difficult to quantify, since no such genetic subgroups have been indisputably identified for MS and related disorders, and examples of subgroup frequencies are purely speculative even within HLA associations. Some evidence comes from differing clinical characteristics and sub-phenotypes, which have been shown to have varying genetic associations in systemic lupus erythematosus (SLE) [12]. The gradient of phenotypes within the disease may be composed of genetic subgroups, or more likely be enriched with some particular subgroup(s), thereby resulting in different associations.

For example, in MS the HLA-DR15 allele is strongly associated to disease (60% carriage rate cases, 30% carriage rate controls) [13] ^,^ [14]. Recent gene network studies have also indicated that different gene networks may show association in DR15+ and DR15− cohorts (data not shown). If a subgroup of MS based on HLA-DR15 exits, it may be reasonable to consider stratification via HLA alleles. Given this assumption, Equation 1.6 can be used to calculate that an increase in sample size of approximately 2.1 is needed to obtain similar association statistics to that of the DR15+ subgroup alone. While all genetic associations are not likely to be perfectly correlated with DR15 status due to the presence of modifier genes [5], division on HLA status may increase power to detect genes which interact with HLA or which act together in DR15− afflicted individuals. The utility of this insight is particularly useful when less obvious alleles than HLA are present within cases and stratification parameters are unknown.

Table 7 shows an empirical example of the impact of a subgroup on the OR, in a hypothetical case/control cohort of 1000 patients and 1000 controls. This demonstrates that an OR of 1.12 (case MAF 0.35, control MAF 0.325) could be skewed by a factor of 1.86 if the data was a result of 20% of the cases representing a subclass (MAF 0.5), with an OR_sub_ of 2.08. This calculation can also be approximated by Figure 3, by taking the second lowest curve (MAF 0.35 in cases). An increase in the subgroup MAF of 0.15 gives a relative increase of 1.86 as observed on the y-axis.

**Table 7:** A hypothetical example illustrating that in data with 1000 cases and 1000 controls, if a SNP had altered frequency only in a subgroup of 200 cases, the OR would be skewed.

Original Data			Adjusted Data		
	Allele 1 (%)	Allele 2 (%)		Allele 1 (%)	Allele 2 (%)
Cases (n = 1000)	700 (35)	1300 (65)	Cases (n = 200)	200 (50)	200 (50)
Controls (n = 1000)	650 (32.5)	1350 (67.5)	Controls (n = 1000)	650 (32.5)	1350 (67.5)
	OR	1.12		OR	2.08

In the example, the allele frequency in the subclass (50%) masks the full effect of association, and moving the remaining cases to controls gives an OR of 2.08 for the SNP in the subclass. This corresponds to an OR error factor of 1.86, which can also be determined by inspecting the second lowermost curve in Figure 3 (case MAF 0.35) with a MAF increase in the subgroup of 0.15.

This corresponds to a *p*-value change from 0.095 (not significance even in a single SNP study) to 2.4x10^−11^ (genome wide significance), even with a drastic reduction in case sample size. This association would assuredly be bypassed due to Type II error. Furthermore, the MAF for cases in the subclass is 0.50, indicating that the SNP could occur in every single case in the subclass as a heterozygote. While an ideal example, many SNPs could act in this fashion while being masked by occurring together in subclasses composed of a low proportion of total cases.

In this example, the increase in samples needed corresponds to the value of γ in equation 1.5. Substituting for a,b,c,d and σ = 0.8 gives γ = 8.24. Therefore, to obtain a similar χ^2^ value without subgrouping, the cohort must be expanded to over 8 times the number of cases and controls (i.e. 8240 cases and 8240 controls).

Examining from the reverse perspective illustrates the impact of underlying subgroups on the *p*-value and OR, in a hypothetical case/control cohort of 1000 patients and 1000 controls, based on heterogeneity percentage (Table 8). When all cases conform to the subgroup, a MAF of 38% in cases compared with 30% in controls reaches the border of genome-wide significance (9.3x10^−8^, OR = 1.43). With increasing heterogeneity, that is, proportion with similar allele frequencies to controls, the *p*-value and OR both decrease accordingly.

**Table 8:** A hypothetical example demonstrating the effect of heterogeneity (cases without the minor allele affecting disease) in data with 1000 cases (MAF 30.0%) and 1000 controls (MAF 38.0%).

	Controls			Cases				
Heterogeneity percentage	MAF%	Allele 1	Allele 2	MAF%	Allele 1	Allele 2	OR	*p*-value
0%	38%	760	1240	30.0%	600	1400	1.43	9.27E-08
10%	38%	760	1240	30.8%	616	1384	1.38	1.64E-06
20%	38%	760	1240	31.6%	623	1368	1.33	2.15E-05
30%	38%	760	1240	32.4%	648	1352	1.28	0.000209
40%	38%	760	1240	33.2%	664	1336	1.23	0.001524
50%	38%	760	1240	34.0%	680	1320	1.19	0.008408
60%	38%	760	1240	34.8%	696	1304	1.15	0.035452
70%	38%	760	1240	35.6%	712	1288	1.11	0.115551
80%	38%	760	1240	36.4%	728	1272	1.07	0.295186
90%	38%	760	1240	37.2%	744	1256	1.03	0.601475

The *p*-value, at the border of genome-wide significance, lowers as the percentage of heterogeneity (with MAF as given by cases) increases.

In order to determine if increased heritability of complex disorders might exist within subgroups, we conducted a simulation of two subgroups of MS by utilizing data from the 123 reported markers from the published meta-analysis, plus HLA [15]. First, we determined the genotypes in our Swedish cohort of 632 cases and 527 controls at the given loci, plus associated HLA alleles. Using the model of So based on multifactorial liability threshold [16], the variability explained by these markers in our cohort was 32%, very close to the reported value of 30.7% in GWAS data [17]. Next, we assumed that the half of data not in each subgroup would have allele frequencies similar to controls, with case allele frequencies adjusted accordingly. Using these conservative subgrouping assumptions, the variability explained within each subgroup averaged 51.2%, even while using half the markers and disease frequency. This indicates that the low variance estimates due to known genetic factors could be, at least in part, explained by inconsistent effects due to subgroups present in complex disease.

Based on our results, at least a portion of the “missing heritability” may be explained by incomplete penetrance of associated markers across disease cohorts due to subgroups or phenocopies. While fine mapping and sequencing may detect low frequency and rare variants contributing to disease, better methods to detect variants present within GWAS, but below detection thresholds, are required. Identification of subgroups of disease through promising approaches such as network and pathway analysis may determine interactions otherwise obscured by noise. New methods to combine low effect markers are required to build up subgroup classification across similar phenotypes.
